# Self-care coping strategies in people with diabetes: a qualitative exploratory study

**DOI:** 10.1186/1472-6823-9-6

**Published:** 2009-02-20

**Authors:** Margaret M Collins, Colin P Bradley, Tony O'Sullivan, Ivan J Perry

**Affiliations:** 1 University of California Cooperative Extension, Tuolumne County, Sonora, CA, USA; 2 Dept of General Practice, University College Cork, Cork, Ireland; 3 Irishtown Health Centre, Dublin, Ireland; 4 Dept of Epidemiology & Public Health, University College Cork, Cork, Ireland

## Abstract

**Background:**

The management of diabetes self-care is largely the responsibility of the patient. With more emphasis on the prevention of complications, adherence to diabetes self-care regimens can be difficult. Diabetes self-care requires the patient to make many dietary and lifestyle changes. This study will explore patient perceptions of diabetes self-care, with particular reference to the burden of self-care and coping strategies among patients.

**Methods:**

A maximum variation sample of 17 patients was selected from GP practices and diabetes clinics in Ireland to include patients with types 1 and 2 diabetes, various self-care regimens, and a range of diabetes complications. Data were collected by in-depth interviews; which were tape-recorded and transcribed. The transcripts were analysed using open and axial coding procedures to identify main categories, and were reviewed by an independent corroborator. Discussion of the results is made in the theoretical context of the health belief, health value, self-efficacy, and locus of control frameworks.

**Results:**

Patients' perceptions of their self-care varied on a spectrum, displaying differences in self-care responsibilities such as competence with dietary planning, testing blood sugar and regular exercise. Three patient types could be distinguished, which were labeled: "proactive manager," a patient who independently monitors blood glucose and adjusts his/her self-care regime to maintain metabolic control; "passive follower," a patient who follows his/her prescribed self-care regime, but does not react autonomously to changes in metabolic control; and "nonconformist," a patient who does not follow most of his/her prescribed self-care regimen.

**Conclusion:**

Patients have different diabetes self-care coping strategies which are influenced by their self-care health value and consequently may affect their diet and exercise choices, frequency of blood glucose monitoring, and compliance with prescribed medication regimens. Particular attention should be paid to the patient's self-care coping strategy, and self-care protocols should be tailored to complement the different patient types.

## Background

Diabetes is both a lifelong and a twenty-four hour a day condition. Glucose control is almost entirely in the hands of the person who lives with this condition. His/her motivation to eat, exercise, take medication, test glucose levels, and maintain a normal body weight all vie with life's other motivations.

One of the biggest challenges for health care providers today is how to address the continued needs and demands of individuals with chronic illnesses like diabetes [1]. Conversely, the challenge for patients is how to obtain the necessary skills to effectively manage their diabetes. Recent research has increased the emphasis on tight metabolic control as several large intervention studies have indicated maintaining good metabolic control can delay or prevent the progression of complications associated with diabetes [2-5]. The introduction of home blood glucose monitors in the 1980's and widespread use of glycosylated haemoglobin as an indicator of metabolic control have also contributed to changes in the approach to diabetes self-care, and thus have shifted more responsibility to the patient [6]. The advancements in technology and recommendations from these studies are very important and convincing but present health care providers with the challenge of implementing them in practice. A recent qualitative study examining self- monitoring of blood glucose in patients with type 2 diabetes suggests the role of the health professional is crucial to patient understanding of their blood glucose fluctuations and whether or not the patient responds to the high blood glucose reading with an appropriate self-care action [7]. Diabetes self-care requires the patient to make many dietary and lifestyle changes simultaneously further emphasizing the need for self-care management support. A meta-analysis of self-management education for adults with type 2 diabetes, reported self-management education improves glycaemic control at immediate follow-up, and increased contact time increases the effect [8]. However, the benefit declines one to three months after the intervention ceases, suggesting that learned behaviours may change over time and continuing education is necessary [8]. Prescribed regimen changes can also impact the patient, as Bradley *et al*. observed: patients reported a higher self-care burden when insulin was added to their regimen [9]. Williams *et al*. found patients who feel their health care provider understands and supports them were more likely to have higher levels of self-confidence resulting in successful behaviour change [10]. The self-care burden is largely the responsibility of the patient as Glasgow and colleagues emphasize; the patient is the one who must decide which diabetes self-care strategies to practice, and ultimately they experience the results of those self-care actions [11]. Furthermore, Ockleford *et al*. suggests health professionals should tailor their patient self-care support based on the degree of personal responsibility the patient is willing to assume towards their diabetes self-care management [12]. Patient preferences for group or private self-care management education should also be taken into consideration [12].

Examining health-related behaviour from a theoretical context may further describe the diabetes self-care decision making process. Several constructs that possibly will help explain the behaviours required for successful diabetes self-care management are: self-efficacy, locus of control, health belief and health value. Self-efficacy is concerned with the perceptions a person has about their ability to perform a task and can influence the acquisition of new behaviours, while locus of control contends with an individual's perception of whether their action determines an outcome or is outside of their control [13,14]. Health belief can be influenced by the person's perceptions of the perceived benefits of positive behaviour change, and health value interacts with locus of control to influence health-protective behaviours [15,16].

The aim of the present study is to explore patient perceptions of diabetes self-care and to identify different self-care coping strategies among patients using qualitative techniques. Discussion of the patient self-care coping strategies and corresponding patient types is made in the theoretical context of the health belief, health value, self-efficacy, and locus of control frameworks to help illustrate the findings within the context of applied health science.

## Methods

A maximum variation sample of 17 patients was selected from GP practices and hospital diabetes clinics in the Irish Southern and East Coast Area Health Board regions, designed to include patients with types 1 and 2 diabetes, various self-care regimens, and a range of diabetes complications. We targeted approximately 50% males; at least 2 people younger than 35 years and at least 2 people older than 70 years; at least 60% with type 2 diabetes; 5 people with a duration of 5 years or less; at least 50% diagnosed with one or more diabetes complication (retinopathy, nephropathy, neuropathy); and at least 2 people following each of the self-care regimens (diet only, oral, insulin, and oral/insulin) (Table 1) [17]. The age range of the patients was from 28 to 74 years, with a mean age of 60 years.

**Table 1:** Patient Types Characteristics

	***Proactive manager***	***Passive Follower***	***Nonconformist***
Type of Diabetes			
*-Type 1*	2	3	2
*-Type 2*	3	5	2

Age **(years)**			
*(mean)* *range*	54 (28–70)	61 (49–74)	63 (47–74)

Duration **(years)**			
*(mean)* *range*	11 (2–23)	13 (1–42)	21 (4–36)

Sex			
*-Male*	3	4	2
*-Female*	2	4	2

Regimen			
*-Diet*	1	2	1
*-Oral*	1	4	0
*-Insulin*	2	2	2
*-Oral/Insulin*	1	0	1

Complications			
*-Retinopathy*	2	0	2
*-Nephropathy*	0	0	2
*-Neuropathy*	1	2	1

### Ethics Approval

Ethics approval was obtained from both the Cork University Hospital (CUH) and the Irish College of General Practitioners (ICGP) Ethics Committees. The study protocol and patient consent forms were reviewed, and approved by both committees.

### Recruitment

Recruitment took place over a six month period. Patients who provided the necessary characteristics required for the maximum variation sample were approached in the Cork University Hospital diabetes clinic by the lead researcher (Research Associate in Dept. of Epidemiology & Public Health, UCC) or were invited by their GP to participate in the study. The study objectives and methods were explained to all patients who were approached. Patients who agreed to participate were asked to give signed informed consent before the in-depth interviews.

### Interview process

Data were collected by tape-recorded, one to one, in-depth open-ended interviews, ranging from one to one-and-a half hours in length. Interviews were conducted by the lead researcher in a private room in the department of Epidemiology and Public Health in Cork or the offices of the Irish College of General Practitioners in Dublin. The interview began with a chat to establish rapport, and a brief clinical history was obtained from each patient including: date of diagnosis; type of diabetes; type of regimen; and any other conditions associated with diabetes [17]. Patients were asked to talk about their diabetes, and describe their daily routine. Then they were asked, "How does diabetes impact your life?" A topic guide developed from the Audit of Diabetes Dependent Quality of Life (ADDQoL) questionnaire, an instrument that measures how the burden of managing diabetes affects different aspects of life was used only if further probing was necessary [18].

### Data analysis

The audio transcripts from the interviews were listened to repeatedly for data familiarization by the lead researcher. Computer software (Annotape) was used to facilitate the transcribing and data coding process by annotating and indexing the original audio interview files and storing segments of tape with an open code that was as close to what the interviewee said as possible. The data analysis was conducted by the lead researcher. The data was examined for similarities and differences to open up ideas and add dimension to the patients' stories [19]. Codes from all of the interviews were sorted in alphabetical order to provide a visual picture of the sample and facilitate grouping into categories. Each individual open code was compared to the rest of the open coded data to establish the categories [20]. Similarities and differences across sub-groups were also explored. Conceptual diagrams were used to show relationships between the emerging themes and their dimensions to add further scope and support to the developing categories or themes [19].

### Data credibility

A senior qualitative researcher in the Department of Epidemiology and Public Health, UCC independently reviewed a random sample of five interviews (including audio tapes, field notes, and coding framework). Written feedback was provided to the lead researcher on coding and provided corroborating evidence of the logic of the decision-making [21]. The methodology and final coding framework was also reviewed by one of the co-authors with extensive knowledge in qualitative research methods (CPB).

## Results

A picture emerged of the different diabetes self-care coping strategies across various patient types (see Table 1) [22-24]. Three major categories were identified and capture a wide range of self-care coping strategies: 1.) **self-care health value **(the value the patient places on diabetes self-care as it relates to his/her health on a continuum from high to low), 2.) **self-care responsibility **(who is responsible for self-care, which varied from complete personal responsibility to reliance on others for some areas such as diet, or denial of responsibility for self-care), and 3.) **self-care coping strategies **(five self-care coping strategies or themes were identified: ***planning ***of diet and exercise activities, ***testing, recording***, ***assessment ***of blood glucose information, and ***adjustment ***of diet and exercise activities).

**Figure 1:**
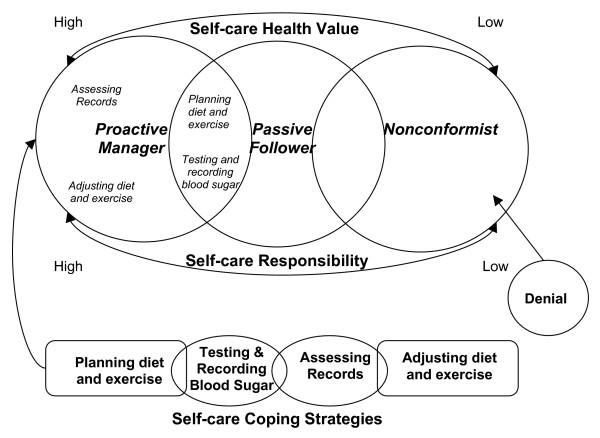
**Diagram of the Relationships Between Categories and Themes**.

Three patient types, using type in Weberian sense [22-24] could be distinguished, which were labeled: 1.) "proactive manager," 2.) "passive follower," and 3.) "nonconformist." Distinct differences were found in self-care coping strategies across the three patient types (see Figure 1). The patient types responses to each theme varied as detailed below:

### Proactive manager

The proactive manger adopts a healthy lifestyle. Many said that they viewed their diabetes as a condition, not a disease, which they had to manage. Although the numbers were small, there were more male proactive managers and most were using insulin or a combination of oral medication and insulin. The three key self-care characteristics of the proactive manager are: 1. high self-care value; 2. acceptance of personal responsibility for most aspects of diabetes self-care; 3. active planning of glycaemic control activities (testing, recording, assessment of blood glucose records), and adjustment of self-care as required (*see patient en vivo quotes below*). A proactive manager values the relationship between self-care and his/her ability to positively influence metabolic control, especially with daily blood glucose readings. A proactive manager takes a proactive approach to their self-care by using such strategies as reading product labels, planning meals, and assessing medication and blood glucose records for patterns. The proactive manager is very assertive with health care professionals and frequently questions things they do not understand, and many would also negotiate self-care treatment goals. However, it should be noted that some proactive managers are comfortable with the more complex self-care strategies, such as matching insulin to food, and some are not as flexible with self-care adjustment.

#### Self-care Health Value

"Since I got the meter for the blood tests, I think that I have managed it better than I used to. When I'm in control, I have peace of mind." (MI1)*

"My general health is a lot better since I have been treating it for diabetes." (MO2)*

#### Self-care Responsibility

"My family all accept the fact that I'm a diabetic, but they all accept the fact that I take care of my diabetes myself, and that if I'm in trouble I would ask them or tell them what I need. Other than that they all depend on me instead of I depending on them." (FO/I3)*

#### Self-care Coping Strategies

##### Planning

"I do plan the meals now and we don't do anything greasy or anything like that." (FD4)*

"I read every label; I must be the greatest label reader in the country." (FO/I3)*

"The one thing that I'm very conscious of is sugar and fat. I'm getting more aware of it, where I'd buy say like tinned stuff and I'd never questioned it, but now I am. The stuff I would take months ago, now I wouldn't take it." (MO2)*

##### Testing, recording and assessment of blood glucose information

"The first thing in the morning, it's a ritual now, the first thing I go to is my meter, and I check my blood with the meter. I would enter that into a diary, so it is recorded in two ways on the meter and in the diary." (MO2)*

"I probably over check, about four times before meals. What I want, you see, is to know what dose I need to take with my meal." (MI5)*

##### Adjustment of self-care

"I was much higher in the beginning, I'd often be eight or nine, but since I have been on a diet and stuck to the diet nothing goes over five or six." (FD4)*

"I feel quite free to ask him questions (doctor), and I find the nurses in the hospital are very helpful, very easy to talk to, and very easy to get information from." (FO/I3)*

"Well I intend to drop in (diabetes daycare centre at the hospital) after the holiday period is over, because I want to get back down to my 1,200 calorie diet and get my sugars down to the single figures." (FO/I3)*

"If my reading was high, I would do the exercise bike for a few minutes." (MI1)*

"If say I'm on holiday, or if I'm doing something that's not normally part of your routine, it's a bit more difficult. You get sugars that are over ten, but that doesn't bother me that much because, that's the reason I check (blood glucose) as frequently as I do. If I do find it over ten, I can always give more insulin, and that's the benefit of doing the rapidly acting insulin regimen." (MI5)*

"I have been on holidays with other people with diabetes, and they said, 'oh I'm having two drinks tonight and I'm going to have a dessert for my dinner, so I'm going to up my insulin tonight.' I feel that would put your control out completely." (FO/I3)*

* Patient Codes: Gender M = Male, F = Female; Regimen D = Diet, I = Insulin, O = Oral; O/I = Oral/Insulin; and patient ID number at the end

### Passive Follower

The passive follower prefers structure to flexibility, with no variation in medication or meal times. Most of the passive followers had type 2 diabetes and used only oral medications. The key self-care attributes that define a passive follower are: 1. consistency in self-care; 2. reliance on others (family or healthcare professionals) for some aspects of self-care; 3. resistance to change once the routine or pattern has been established (*see patient en vivo quotes below*). The passive follower prefers consistency in their daily routine in order to cope with the burden that diabetes self-care places on their life. A characteristic that most of the passive followers shared was keeping blood glucose records. It should be noted, however, that the passive follower does not assess or change, his/her self-care independently, even if blood glucose readings indicated there is a problem. Passive followers want to fit into the ideal of the "perfect patient," one who conforms to the "ideal standards," even if this means forgoing an activity. However, passive followers will admit to breaking their prescribed dietary regimen some of the time.

#### Self-care Health Value

"It makes life simpler to kind of follow what you're supposed to be doing anyway." (FO6)*

"I stay fairly constant and fairly level. It's only when you change the routine that maybe things go out of sync." (MI7)*

#### Self-care Responsibility

"As I say I have complete trust in him (GP). Whatever he says, I abide by it, or I try. He seems to have it covered, and as I say, I trust him." (MO8)*

"The GP would make me more aware if there was an increase in my problem. As I say with the GP, he would say, 'we have to watch those figures. Your weight, you have put on weight,' he would make me aware of all of these things." (MO8)*

"Oh my wife does the cooking. My wife knows better than I do. I think she knows what I'm supposed to have." (MO9)*

"You're watching it all the time. I suppose you can't do lots of things that you would want to do, even in the line of walking or gardening." (FI10)*

"Like you're not suppose to have chips now or anything like that, but sometimes I'd have chips, because I think I deserve a little treat, my life is boring enough as it is." (FO11)*

#### Self-care Coping Strategies

##### Planning

"I went through my diet with the dietician. Mondays to Fridays very regimental, the amounts are the exact same (food portion sizes), and the units of insulin have never changed." (MI7)*

"All that I have done is cut out all of the sugars. I don't take any sugar. I don't take cakes, biscuits or anything like that. If I want something as a snack, I take a piece of fruit." (MO8)*

##### Testing and recording of blood glucose information

"About four times a week I usually take my blood sugar and it's in the range. I still keep a diary (blood glucose records). I'm up to the ninth diary since I got diabetes." (MD12)*

"You can see where on your diary, if it keeps within the regular, I know they say four to seven, but I suppose if it's still under 10 it isn't too bad. I actually said to the doctor, there was one day, and he was looking at the diary, and it was 11 something when I went to bed, but it was still eleven in the morning. You know, for no apparent reason, something like that can happen, but he said all the rest are quite ok, they are well within the acceptable range. So he said I wouldn't worry too much about an occasional thing like that." (FD13)*

*Patient Codes: Gender M = Male, F = Female; Regimen D = Diet, I = Insulin, O = Oral; O/I = Oral/Insulin; and patient ID number at the end

### Nonconformist

The last patient type, the nonconformist, did not adopt a healthy lifestyle. They do not follow many of the activities of their self-care regimen, especially prescribed dietary and activity changes. Most of the patients in the nonconformist group were using insulin or a combination of oral medication and insulin. Key characteristics for the nonconformist are: 1. lack of personal responsibility for diabetes self-care; 2. low self-care health value; 3. lack of planning, assessment, or adjustment of self-care (*see patient en vivo quotes below*). A nonconformist does not feel responsible for their self-care, and is often in denial about how diabetes will affect their future health. A nonconformist does not place much value on self-care and some even admit that they know what they are supposed to do but can't be motivated to make changes. A nonconformist would not actively plan meals or exercise, test blood sugar, or assess their self-care routine. Conversely, even though the nonconformist does not follow his/her dietary regimen and does not test blood sugar, he/she will admit to taking their medications.

#### Self-care Health Value

"I just don't change easily, I couldn't be bothered, I suppose. I'm quite happy with what is bad for me. I still go ahead at times and eat things that I know I shouldn't be eating." (MI14)*

"I said to him (doctor), 'You'll get as much information from me as you would from my diary as there is nothing in it." (FO/I15)*

#### Self-care Responsibility

"Maybe I'll develop some complications of diabetes, eye problems you know, kidney problems, what will happen in the future will happen." (MI14)*

"Diabetes has no effect on my life. It has no effect on me to the extent, as I say it was just by chance that it was diagnosed. It could have been there for years. I could have gone for two or three more years with diabetes without knowing it." (MD16)*

#### Self-care Coping Strategies

##### Planning, testing and assessment

"Sometimes I have the awful habit of feeding everyone and leaving myself to last, and then you know that you have to have it (food), and when that occurs you're inclined to shoving it down, and then the sugars go up." (FI17)*

"I'd only walk around if it was fine weather and if I was to go from A to B. If I could walk there that's fine, but don't ask me to be going for a walk. Do you know what I mean?" (FI17)*

"I don't test at all (blood sugar testing). I haven't tested in awhile, because my meter, either the battery is gone or something like that, and I haven't got time to get another meter." (MD16)*

"If I want to get something done I will set myself targets, but as for targets again where my diabetes is concerned, not really. I just can't seem to be motivated to do anything about my diabetes." (MI14)*

* Patient Codes: Gender M = Male, F = Female; Regimen D = Diet, I = Insulin, O = Oral; O/I = Oral/Insulin; and patient ID number at the end

## Discussion

Patients' perceptions of their self-care varied on a spectrum, displaying differences in self-care responsibilities such as competence with dietary planning, testing blood sugar and regular exercise. The prescribed regimen and to a lesser extent gender may also account for some the differences across the patient types. To help describe our patient's self-care coping strategies we will use the health belief, health value, self-efficacy, and locus of control frameworks.

### Proactive Manager

A proactive manager is a patient who independently monitors blood glucose and adjusts his/her self-care regime to maintain metabolic control. Although the numbers were small, it is important to acknowledge there were more male proactive managers and most were using insulin or a combination of oral medication and insulin. Previous studies have reported that men with diabetes experience less disease impact and more treatment satisfaction than women possibly due to the different roles that men and women occupy in society [25-27]. Women have multiple role responsibilities and may find the diabetes regimen difficult to fit into their busy lives [28]. Men on the other hand, are typically more narrowly focused with their roles and responsibilities and possibly less likely to let the diabetes regimen interfere with their life [29].

Proactive managers believe their self-care is successful which is consistent with one aspect of the health belief model; belief of treatment effectiveness [30]. They mentioned the feedback they received from their blood glucose readings told them how they were doing; therefore, testing, recording and assessing their blood glucose were self-care coping strategies that gave them some reassurance about the management of their diabetes self-care. Acceptance of personal responsibility for most aspects of diabetes self-care was another attribute that the proactive managers shared and is consistent with the construct of internal locus of control where an individual believes his or her own self-care behaviour determines or influences an outcome [31]. The proactive manager accepts a high degree of responsibility for their health and believes there self-care actions will positively influence their metabolic control. The combination of personal self-care responsibility and high health value may explain why the proactive manager engaged in so many health-protective behaviours [15].

The inclusion of insulin in the prescribed regimen of most of the proactive managers may trigger more complex glycaemic control behaviours like assessment of blood glucose records and adjustment of self-care in some individuals. The proactive manager's ability to self-regulate or assess and adjust patterns of self-care is a coping strategy that sets them apart from the passive followers and the nonconformists. Bandura found that the strength of individuals' self-efficacy or belief in their ability to perform a behaviour is directly related to how they cope with the new behaviour and may also impact their willingness to perform additional health-protective behaviours [13]. Previous work in self-efficacy has consistently found that diabetes self-care behaviours are task specific or independent of one another, for example, an individuals' perceived capability to take their medication may be different from their perceived capability of testing their blood sugar [32]. The proactive manager's explanations of their self-care coping strategies indicate they have mastered many different self-care tasks and are operating with a high degree of personal self-efficacy compared to the other patient types. Proactive managers enjoyed greater flexibility with meal times and food choices due to their coping strategies of pattern assessment and adjustment which allowed them to maintain metabolic control with more personal freedom. However, it should be noted there is some controversy surrounding the extent of independent patient self-care regulation. A recent study has called this proactive approach to self-care regulation "strategic non-compliance" which sends the wrong message to patients and providers with the label of "non-compliance" [33]. Tattersall argues patients must have the freedom and approval from their providers to change their treatment, specifically patients with diabetes should feel empowered to make adjustments in their insulin dose to match their food choices [34].

### Passive Follower

A passive follower is a patient who follows his/her prescribed self-care regime, but does not react autonomously to changes in metabolic control. The passive follower does not share the proactive manager's propensity for flexibility and prefers structure with no variation in medication or meal times. Most of the passive followers had type 2 diabetes and used diet or oral medications to control their diabetes. Passive followers tended to rely more on powerful others to help them make self-care decisions, such as a partner or family member for help with their diet, or the health professional for help with their self-care regimen; following the regimen was very important to the passive follower. This behaviour is consistent with external locus of control where individuals believe their health may be controlled by outside forces that are independent of their actions [14]. Parry and colleagues also found when patients identified the main cause of their condition as outside of their control like genetic factors; they placed responsibility for disease management with health professionals [35]. Furthermore, the passive followers reliance on powerful others may have indirectly affected their health value and in turn negatively influenced their adoption of health-protective behaviours like making changes to their lifestyle when their blood glucose readings indicated there was a problem. Since most of the passive followers were on diet only or oral medications, they may not be aware how to react to blood glucose problems, unlike the proactive managers who are able to match insulin to food or make lifestyle changes when their assessment of blood glucose records imply self-care change is needed. This finding is important for both patients and health professionals, as passive followers may need more support to help them make appropriate lifestyle changes, especially when their blood glucose readings show corrective action is needed. It has been noted in the literature both internal and external powerful other health locus of control were associated with regimen compliance using the Multidimensional Health Locus of Control scales [36]. The fact that passive followers may be regiment compliant should not imply they do not need further training to adopt new behaviors which would allow them to make self-care changes independently. The passive followers may benefit from self-care decision support. There is real opportunity for the passive follower to acquire more health-protective behaviours which may help them to maintain better metabolic control.

### Nonconformist

The nonconformist is a patient who does not follow most of his/her prescribed self-care regimen. The nonconformists felt things like their future health were not within their control; their coping strategy was one of denial. This finding concurs with Parry *et al*. who identified a similar group of patients (labeled as 'up to them') who regard the cause and management of their condition as outside their control [35]. The nonconformist did not follow many of the activities of their self-care regimen, especially prescribed dietary and activity changes. Since the nonconformist believed their future health was outside of their control they did not engage in health-protective behaviours which is linked to low self-care health value. The nonconformist accepted minimal personal responsibility for their self-care which may indicate they did not believe preventative health actions like positive lifestyle changes would impact their future health [37]. Many of the nonconformists were in denial of the seriousness of their condition, which may also explain why they suffered from more complications than either the passive followers or the proactive managers. It is interesting to note, like the proactive managers, most of the nonconformists were on insulin or a combination of oral medications and insulin. Previous work has shown insulin treated patients reported the most negative diabetes impact compared to patients on oral medication or diet only regimens [38]. The nonconformist may believe that the burden of diabetes is insurmountable, and that adopting positive lifestyle behaviours is too difficult. Previous work however suggests that the nonconformist may benefit from group education where they are exposed to other patient's self-care success stories [39].

### Deviation from patient type

It is important to note, the patient types were not static. No patient is a perfect fit or stays within type all of the time. The patient type is an analytical construct that allows us to ascertain similarities as well as deviations in typical course of conduct or, in the case of diabetes self-care, typical self-care coping strategies by patient type [23]. Passive followers, more than any of the other patient types, want to fit into the ideal of the "perfect patient," following his/her prescribed self-care regimen and not taking action even when fluctuations in blood glucose indicate the need for change. Staying within type is not always possible. The passive follower will admit to breaking his/her prescribed dietary regimen, and not all proactive managers are comfortable operating with the more complex self-care strategies, such as matching insulin to food.

### Study limitations

It is important to note the limitations of this study. The study sample was heterogeneous and included both patients with types 1 and 2 diabetes which may account for some of the differences in coping strategies. The qualitative data was not longitudinal; therefore, no inferences can be made about behaviour changes that occurred over time. There was some disconfirming evidence among the patient types regarding the decision making process used to adopt new health-related behaviours that should have been probed in further interviews. However, this was not possible due to time and resource limitations. No one theory alone captured the range of patient self-care coping behaviours or strategies. All three theories in combination provide a much more complete picture of how people with diabetes cope with the burden of their self-care.

## Conclusion

Patients have different diabetes self-care coping strategies which are influenced by their self-care health value and consequently may affect their diet and exercise choices, frequency of blood glucose monitoring, and compliance with prescribed medication regimens. Thus, assessments and interventions should be comprehensive and integrate available theories for studying human behaviour to capture the full range of diabetes self-care behaviours. Particular attention should be paid to the patient's self-care coping strategy and self-care protocols should be tailored to complement the different patient types. Counseling on self-care coping strategies may be beneficial to patients with poorly controlled diabetes or those patients who want more flexibility in their self-care regimen. Gender, type of diabetes, and regimen should also be considered when counseling patients on self-care management. The findings from this study may be relevant to the design of quantitative instruments addressing self-care coping strategies in patients with diabetes and other chronic conditions.

## Competing interests

The authors declare that they have no competing interests.

## Authors' contributions

MMC was involved in the study at the conceptual stage including: funding, ethics approvals, and managed study recruitment, data collection, data management, data analyses, and manuscript write-up. IJP conceived the study, obtained funding, supervised MMC on all aspects of the study, and helped write the manuscript. CPB contributed to the qualitative methodology, data analysis, and manuscript write-up. TOS contributed to the clinical input.

## Pre-publication history

The pre-publication history for this paper can be accessed here:


